# A late presenting left‐sided congenital diaphragmatic hernia repair complicated by postoperative chylothorax: A case report

**DOI:** 10.1002/ccr3.3528

**Published:** 2020-11-11

**Authors:** Sandesh Raj Upadhaya, Utsav Joshi, Sagar Gyawali, Bijay Thapa, Anupama Thapa

**Affiliations:** ^1^ Department of Pediatric Surgery Kanti children's Hospital Kathmandu Nepal; ^2^ Department of Internal Medicine Rochester general hospital New York NY USA; ^3^ Department of Internal Medicine Tribhuvan University Teaching Hospital Institute of Medicine Kathmandu Nepal

**Keywords:** chylothorax, congenital diaphragmatic hernia, conservative, left‐sided, postoperative

## Abstract

Management of chylothorax after repair of late presenting congenital diaphragmatic hernia is debatable. Conservative management in the form of close monitoring of chylous output with nutritional support appears convincing to surgery.

## Graphical abstract

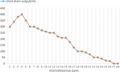

## 1 INTRODUCTION

Chylothorax is a well‐recognized but uncommon complication during the postoperative period following the repair of left congenital diaphragmatic hernia. Conservative management with close monitoring of chylothorax output and patient status seems superior to surgery in managing this complication.

Congenital diaphragmatic hernia (CDH) occurs in approximately 1 in 2500‐3000 live births, with the late presenting CDH comprising 5‐25 percent of the total cases.^1^, ^2^ Late presenting form of CDH has a better outcome if identified and repaired compared to the neonatal CDH. This is due to its less frequent association with other congenital anomalies and mild pulmonary hypoplasia and hypertension.^3^, ^4^ Repair may be complicated by chylothorax, an uncommon issue whose exact etiology is unknown. Multiple studies have shown that chylothorax secondary to direct trauma to the lymphatic vessels can be treated medically with total parenteral nutrition (TPN) and medium‐chain triglyceride (MCT) diet along with intercostal drainage of the effusion. Based on these findings, conservative management appears to be the first option for postoperative chylothorax.^5^, ^6^

Here, we report successful treatment of postoperative chylothorax following the repair of late presenting CDH with intercostal drainage, broad‐spectrum antibiotics, fluid supplementation, and albumin infusion, followed by low‐fat and high‐protein diet until resolution.

## 2 CASE PRESENTATION

A 6‐year‐old female child diagnosed with CDH at 1 month of her life was brought to the outpatient department of our hospital to evaluate her congenital condition.

One month after birth, the child developed difficulty breathing. This was not associated with grunting, intercostal or subcostal retraction, fever, inability to feed, vomiting, drowsiness, or convulsions. For this complaint, she was taken to a local hospital for further evaluation. A plain radiograph of the chest revealed air‐filled loops of intestines in the left lung field. A diagnosis of left‐sided congenital diaphragmatic hernia was established, and the patient was managed conservatively for respiratory distress. Since then, the patient remained asymptomatic and was eventually lost to follow‐up.

On admission to our hospital, the child was completely asymptomatic. Clinically, her general condition was fair, and she was playful. Her respiratory rate was 34/min, pulse was 98/min, and the temperature was 38.4°C. There was no pallor, icterus, cyanosis, edema, palpable lymph nodes, or dehydration. The abdomen was scaphoid‐appearing, and bowel sounds were heard over the left lung field. The point of maximum cardiac impulse was 3 cm below and medial to the nipple.

**FIGURE 1:**
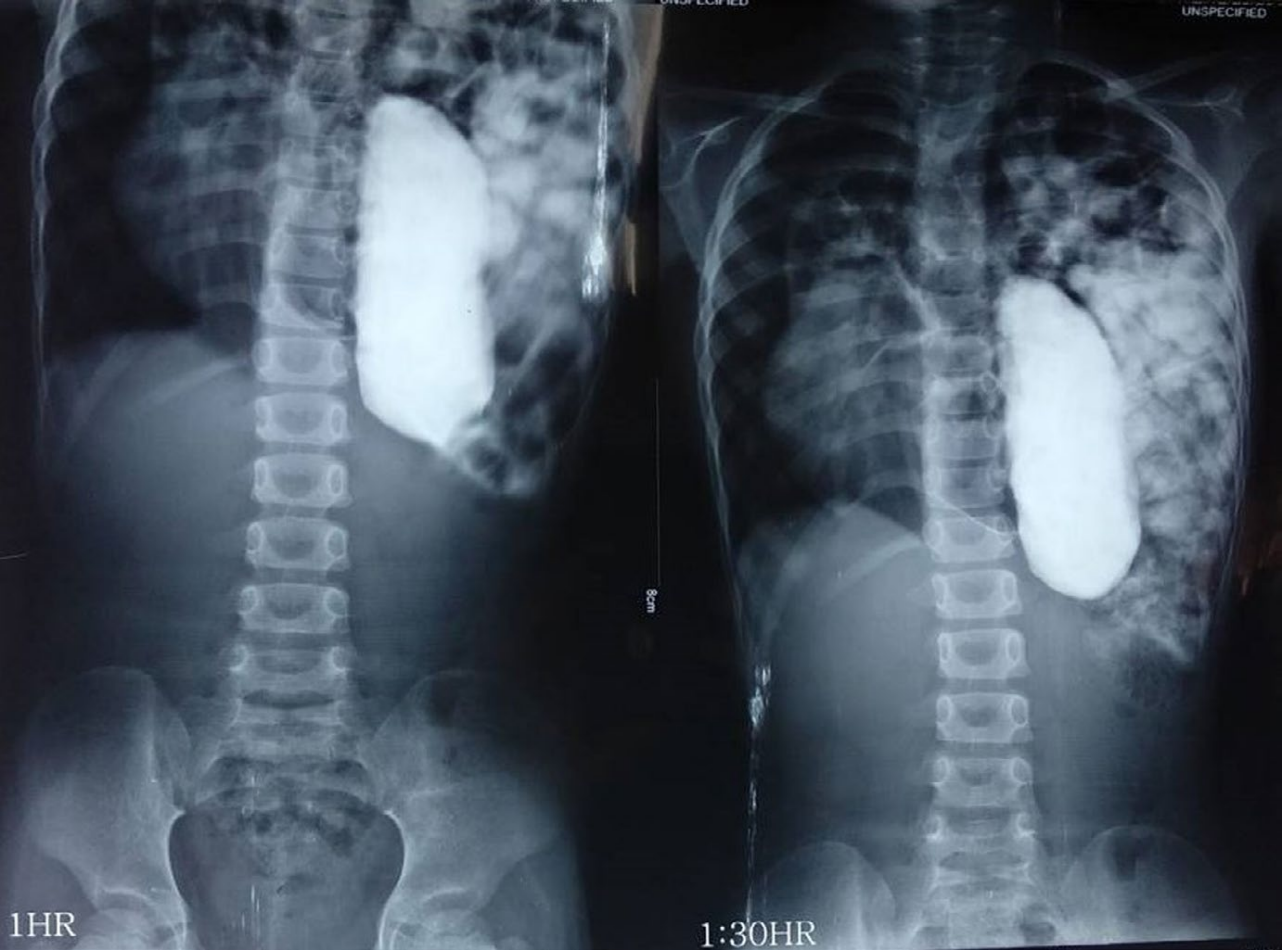
Barium follow‐through showing the presence of stomach and small bowel loops in the left hemithorax

Blood investigation showed: hemoglobin 12.2 gm/dL, white blood cells 5400/mm^3^ (lymphocytes 67%, neutrophils 30%), and platelets 210 000/mm^3^. Her renal function tests showed: urea 3.4 mmol/L and creatinine 26.52 µmol/L. Blood sugar level, liver function tests, serology, and urine routine and microscopic examination were normal. Barium follow‐through study showed the presence of stomach and small bowel loops in the left hemithorax (Figure 1). Contrast‐enhanced computed tomography (CECT) scan of the chest and abdomen revealed a breach in the posterolateral aspect of left hemidiaphragm measuring approximately 5 × 3.2 cm. Intraabdominal contents (small and large bowel, spleen, stomach, mesentery, and mesenteric vessels) were also noted in the left hemithorax leading to a decrease in the abdominal volume and a mediastinal shift to the right. All the imaging findings were suggestive of diaphragmatic hernia, Bochdalek type. The preoperative echocardiographic finding showed normal pulmonary artery pressure.

**FIGURE 2:**
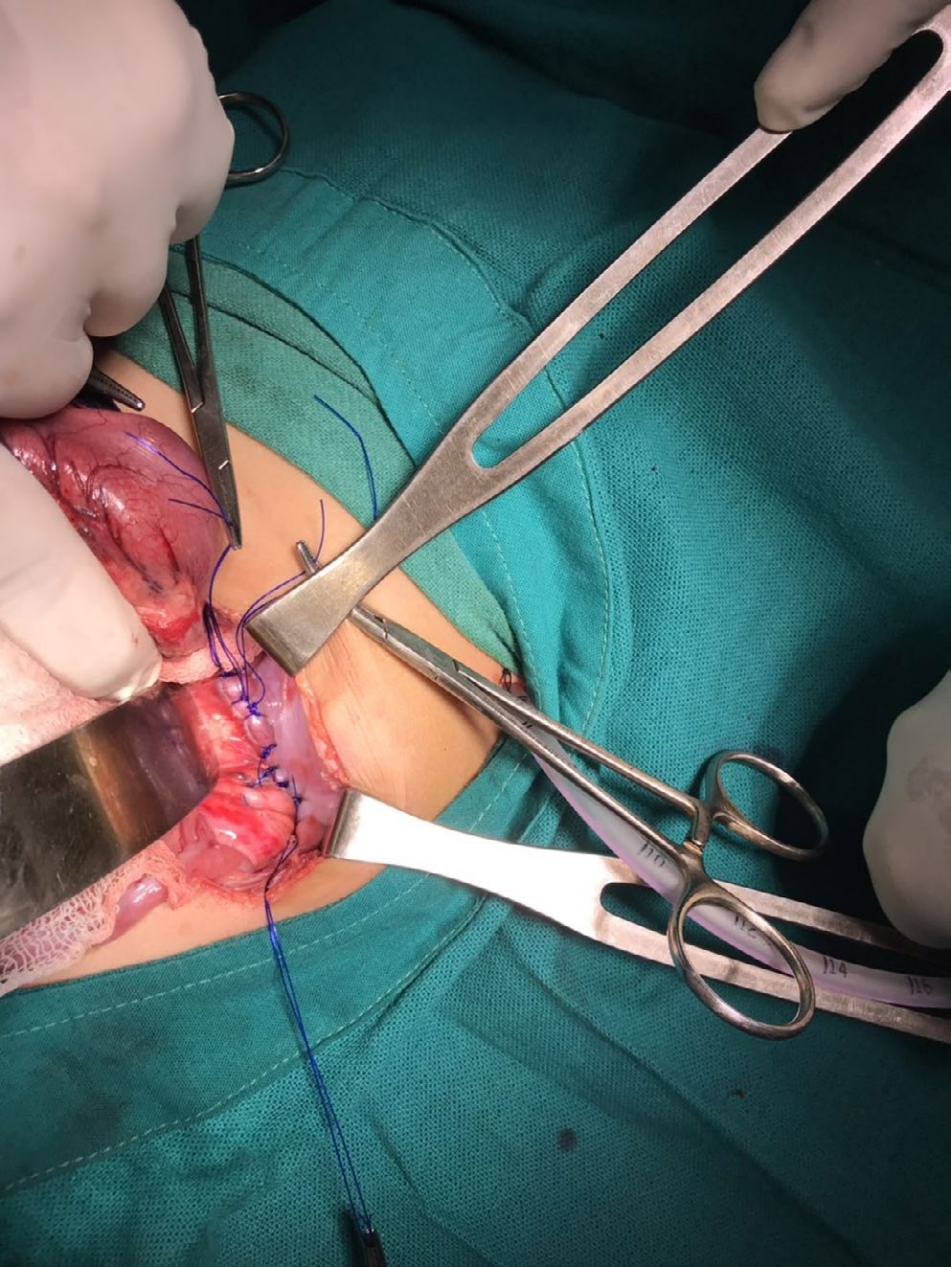
Intraoperative image showing reduction of intrathoracic contents back into the abdomen and suturing of the diaphragmatic defect

The patient was planned for laparotomy. The operative finding was a 6 × 4 cm defect in the left dome of the diaphragm and herniation of intraabdominal contents (stomach spleen, part of jejunum, and transverse colon) without a sac. The intrathoracic contents were reduced back into the abdomen, and the defect was sutured (Figure 2). An intercostal chest tube (size 14 French) was also placed in situ intraoperatively.

**FIGURE 3:**
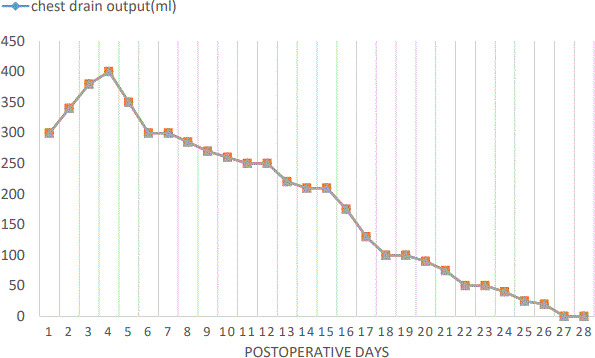
Line graph showing daily postoperative chest drain output

She was admitted to the surgical intensive care unit (SICU) and kept under mechanical ventilation (volume control synchronized intermittent mandatory ventilation mode) for one postoperative day and empiric antibiotics (cefotaxime and metronidazole). She had one spike of fever on the third postoperative day following which her antibiotics were changed to meropenem. Chest tube drainage showed a maximum daily output of 350‐400 mL (approximately 26 mL/kg/day) of straw‐colored fluid for the first seven postoperative days. The daily postoperative chest drain output is shown in a line graph (Figure 3). On the eighth postoperative day, the fluid turned milky white. The pleural fluid analysis showed: lymphocytic predominance (90%), triglycerides 210 mg/dL, and cholesterol 23 mg/dL. With a diagnosis of postoperative chylous pleural effusion, the patient was kept nil per os for three more days. She was treated with intravenous 20% albumin infusion and antibiotics (meropenem and metronidazole). The volume of the chest tube drainage subsequently decreased to 250 mL/day. From the 11th postoperative day, she was kept on low‐fat and high‐protein diet. By the 21st postoperative day, the fluid output decreased to 75 mL/day. On the 27th postoperative day, the chylothorax output decreased to 20 mL/day, and hence, the tube was clamped for 2 days. On the 29th postoperative day, the chest tube was finally removed. By then, her appetite returned to normal, the wound looked healthy, and she was eventually discharged on the 31st postoperative day.

## 3 DISCUSSION

Bochdalek hernia, a form of CDH, results due to a defect in the pleuroperitoneal membrane with herniation of the abdominal contents.^7^ Baglaj et al reviewed the details of 362 children with late presenting CDH and observed that the left posterior hernia was the dominant type found in 79.4% of the CDH patients, the male to female ratio was nearly 2:1 in both the left‐ and right‐sided CDH, and the true hernia sac was found only in 32.7% of the patients.^2^ Patients with late presenting CDH may remain completely asymptomatic and the diagnosis may be incidental, or they may present with acute or chronic gastrointestinal symptoms or less commonly, respiratory symptoms.^8^ The presence of a hernia sac has been suggested as one of the risk factors for the development of postoperative chylothorax. The study by Kavvadia et al showed that two out of three infants who had hernia sac developed postoperative chylothorax, and it was most likely because of the injury to the lymphatic vessels when the sac was excised.^9^, ^10^ However, hernia sac was not evident in our patient. Even in the absence of a sac, vigorous perioperative manipulation and subsequent trauma to the lymph vessels related to the diaphragm may have resulted in chylothorax in our patient.

The diagnosis of chylothorax is made if the drained fluid is whitish, and the value of triglycerides in the fluid is >110 mg/dL.^5^ In the presented case, the drained fluid was milky, and the value of triglycerides in the pleural fluid was 210 mg/dL. In case of any doubt or uncertain diagnosis, the intake of a meal rich in fats by mouth or nasogastric tube results in a dramatic change in the color of the pleural fluid confirming the diagnosis of chylous effusion. Also, pleural fluid leukocyte count comprising more than 90% lymphocytes is a useful and independent marker of chylous effusion,^6^ and the pleural fluid analysis in our patient also yielded a similar finding. Our case report highlights the fact that effusion that occurs earlier may remain colorless for a few days. Clear and light yellow colored chyle may be observed if the patient has not been properly fed.^11^ With time and gradual introduction of enteral feeds, it turns milky. Hence, for effusions that remain persistent, chylothorax should be one of the differential diagnoses, even if the fluid appears clear.

There is no consensus on whether to manage postoperative chylothorax with conservative management or surgery for a particular value of intercostal drain output.^12^ Stringel et al^13^ suggested mass ligation of the thoracic duct just above the diaphragm for chylous effusion >15mL/kg/day, whereas other reports favor surgery only for chylous effusion >100 mL/kg/day.^14^ Hence, keeping aside the absolute value of chest tube drainage, patient management should be based on clinical judgment.^15^ Conservative management in the form of continuous chest tube drainage and cessation of enteral feeds for some time to decrease the lymphatic production followed by nutritional support in the form of TPN and MCT diet and the use of octreotide along with prophylactic antibiotics for hastening the resolution of chylous effusion have been suggested in the literature.^5^, ^10^, ^12^, ^15^, ^16^, ^17^, ^18^, ^19^ Casaccia and colleagues found thoracic lymphatic vessel damage to be the major cause of chylothorax after CDH repair, and chylothorax of such etiology resolved medically in their entire study cohort. The duration for resolution of chylothorax ranged from 4 to 34 days, and the mean duration of TPN in this study cohort was 13 days.^17^ Those who fail to respond to conservative management with a persistently increasing chest tube drainage should undergo some form of surgery (thoracic duct ligation, pleurodesis, and placement of pleuroperitoneal shunt).^5^, ^15^

Postoperative chylothorax following CDH repair may be complicated by infection. There is a significant risk of hypoproteinemia and leukopenia due to the loss of chyle. However, a study by Allen et al showed no correlation between the circulating lymphocytes and the occurrence of an infectious complication. Hence, antibiotics should not be used based solely on the lymphocyte count.^15^, ^20^ In our case, the patient did not show any infectious complication except for one spike of fever as she was kept on prophylactic antibiotic empirically. She had a good appetite and weight gain.

There was no significant difference in the rate of survival among patients with chylothorax vs no chylothorax. However, the length of hospital stay and the number of days on a ventilator are significantly increased in patients with chylothorax.^15^ We decided to choose the conservative modality of management in this patient who had a chylous effusion output up to 26mL/kg/day in the form of continuous chest tube drainage, broad‐spectrum antibiotics, intravenous albumin infusion, and nil per os for 3 days, followed by a high‐protein and low‐fat diet. Eventually, the chylothorax in our patient resolved without operative intervention in 4 weeks.

## 4 CONCLUSION

We report a case of successful conservative management of postoperative chylothorax following the repair of late presenting CDH. The treatment of choice for postoperative chylothorax is debatable. However, our case report highlights that chylous output up to 26 mL/kg/day can also be treated conservatively. Surgery needs not to be the first‐choice option for the management of postoperative chylothorax.

## CONFLICT OF INTEREST

The authors state that there is no conflict of interest.

## AUTHOR CONTRIBUTIONS

SRU: involved in conception and design, collection of data, drafting of the manuscript, and final approval of the version to be published. UJ and SG: involved in the conception and design, drafting of manuscript, revision, and final approval of the version to be published. BT and AT: involved in the management of case, revision of manuscript, supervision and final approval of the version to be published.

## ETHICAL APPROVAL

Ethical approval was not required.

## CONSENT

Informed consent was received from the patient.

## Data Availability

The authors confirm that the data supporting the findings of this study are available within the article.
